# Understanding Jordanian Psychiatric Nurses' Smoking Behaviors: A Grounded Theory Study

**DOI:** 10.1155/2013/370828

**Published:** 2013-06-16

**Authors:** Khaldoun M. Aldiabat, Michael Clinton

**Affiliations:** ^1^School of Nursing, University of Northern British Columbia, 3333 University Way, Prince George, BC, Canada V2N 4Z9; ^2^Rafic Hariri School of Nursing, American University of Beirut, Riad El-Solh, Beirut 1107 2020, Lebanon

## Abstract

*Purpose*. Smoking is prevalent in psychiatric facilities among staff and patients. However, there have been few studies of how contextual factors in specific cultures influence rates of smoking and the health promotion role of psychiatric nurses. This paper reports the findings of a classical grounded theory study conducted to understand how contextual factors in the workplace influences the smoking behaviors of Jordanian psychiatric nurses (JPNs). *Method*. Semi-structured individual interviews were conducted with a sample of eight male JPNs smokers at a psychiatric facility in Amman, Jordan. *Findings*. Constant comparative analysis identified *becoming a heavy smoker* as a psychosocial process characterized by four sub-categories: normalization of smoking; living in ambiguity; experiencing workplace conflict; and, facing up to workplace stressors. *Conclusion*. Specific contextual workplace factors require targeted smoking cessation interventions if JPNs are to receive the help they need to reduce health risks associated with heavy smoking.

## 1. Introduction

Smoking cigarettes is common practice among patients and staff in mental health services throughout the world [1]. Although few studies have assessed smoking behaviors among psychiatric nurses in different countries, their results reported indicate a high prevalence rates compared to those for nurses in other specialties. A literature review by Storr et al. [2] found that psychiatric nurses have higher smoking prevalence rates than nurses working in administration, emergency rooms, medical care, critical care, and gerontology. Psychiatric nurses in the United States are 2.4 times more likely to smoke cigarettes than nurses in other specialty areas [3]. The prevalence of smoking among mental health nurses in the United Kingdom was reported as 17.4% [4]. Two years later the reported prevalence rate was 35% [5] double that reported in the earlier investigation. in the 1980s the smoking the prevalence rates for psychiatric nurses in the United States was reported as 28.6% [6]; almost 14% less than that of 42.4% reported for Great Britain [7].

The high prevalence of smoking among psychiatric nurses threatens professional values (not to harm patients) and delivery of quality services, including patient education, if left unstudied. Furthermore, to neglect the high smoking prevalence rates among psychiatric nurses is to ignore an international agreement about the importance of a health promotion role for all health professionals [8] and to deny psychiatric nurses a legitimate role in health promotion [9]. The health promotion role fits well with the pride psychiatric nurses take in providing holistic care to meet patients' interrelated physical and mental health needs. However, psychiatric nurses who smoke have not yet acknowledged smoking reduction as one of their primary goals for patients, despite the opportunities they have for helping patients cut down on the number of cigarettes they smoke or to stop smoking altogether [9–12].

All the studies referred to in this introduction were conducted in Western countries. Hence, contextual workplace factors that influence smoking behaviors among psychiatric nurses in Arabic speaking countries, including in Jordan, have not been investigated or reported. Therefore, the purpose of this paper is to report the findings of a classical grounded theory study conducted to understand how contextual workplace factors influence the smoking behaviors of JPNs. We intend that our study will encourage other researchers to investigate the relationship between contextual workplace factors and the smoking behaviors of nurses in Arabic speaking countries. Such research is needed to better understand how to help nurses take better care of their health while addressing the smoking reduction and smoking cessation needs of their patients. 

## 2. The Method

This section summarizes the research methods used in the study. A more detailed account can be found in Aldiabat and Clinton [13]. In essence, we used a classical grounded theory approach [14] to investigate how social, psychological, organizational, personal, and cultural factors influence JPNs to become heavy smokers. The study was conducted in Amman, Jordan between 2009 and 2010 following ethical committee approval in Canada and Jordan. Data were collected from a theoretical sample of eight male psychiatric nurses smokers at a psychiatric hospital. Semi-structured interviews, nonparticipant observation, sociometry and ethnographic field notes were used in the study. The constant comparative method of analysis was applied throughout the study. Thus, data collection, coding, and analysis occurred simultaneously. It was found that for JPNs, *becoming a heavy smoker *is a component of a longer process theorized as “*contextualizing smoking behavior over time*.” Four phases are fundamental to this process: (1) becoming a novice smoker; (2) becoming a regular smoker; (3) becoming a heavy smoker; (4) becoming an exhausted smoker. Throughout the study, care was taken to meet an acceptable standard of trustworthiness by fulfilling requirements for credibility, transferability, dependability, and confirmability [13].

## 3. Findings

### 3.1. Becoming a Heavy Smoker

This phase in the *contextualizing smoking behavior over time *psychosocial process explains how Jordanian psychiatric nurses transition from regular smoking to heavy smoking. The eight nurses in this study regarded themselves as regular smokers if the smoked 12–14 cigarettes on most days. They regarded themselves as heavy smokers if they smoked more than 14 cigarettes every day. The participants reported four contextual workplace factors that foster and maintain their habit of heavy smoking: (a) accommodating workplace challenges, (b) living in ambiguity, (c) experiencing workplace conflict, and (d) Facing up to workplace stressors.

### 3.2. Accommodating Workplace Challenges

The eight JPNs attributed heavy smoking to challenges in the workplace. The following verbatim statements describe challenges that influence their smoking.

#### 3.2.1. Normalized Smoking

The participants regarding smoking in the psychiatric workplace as a normal and natural behavior. They distinguished between two kindes of normalization: institutionalized normalization and individualized normalization.

*Organizational Normalization*. According to the participants, organizational normalization of smoking has three salient characteristics:

- widespread tolerance of smoking in the organization: “As you see, smoking is not something strange in this organization” (Yasser);
- regular smoking among psychiatric patients and staff “Both patients and staff smoke freely in [name of psychiatric hospital] which means smoking is an acceptable behavior” (Osama);
- institutionalized availability of cigarettes: “Here, it is not uncommon to find cigarettes everywhere; (...). Sometimes, we miss some medications or equipment, but it is impossible to be without cigarettes” (Ismael).

*Individualized Normalization. *The three most salient charactristics of individualized normalization are:

- smokers and nonsmokers alike accept smoking as a common everyday activity: “I do not feel others [smokers and/or nonsmokers] perceive us differently (...). We are like other people who smoke in Jordan” (Mustafa);
- distributing cigarettes to patients is an integral part of the role of psychiatric nurses: “You know distributing cigarettes to patients is one of our roles, but accepting that role means we [nurses] normalize smoking...” (Kamal);
- none of the participants expressed dissonance associated with smoking. Smokers smoke freely in the presence of non-smoking friends and direct supervisors; although they know that is a clear contravention of Ministry of Health prohibitions.

#### 3.2.2. Challenges That Encourage Smoking

JPNs offer several justifications smoking in the workplace: finding respite from work; managing self-perceptions, including shoring up self- esteem and promoting feelings of personal well being; making time at work go more quickly; rewarding oneself for small achievements; rewarding patients for good behavior; and, controlling them effectively.

 Smoking promotes feelings of comfort and relaxation; I smoke at [name of psychiatric hospital] because smoking gives me more breaks. It helps me treat my negative self-perceptions and boosts my self-esteem. When I smoke I feel my personality becomes stronger (...). Actually, this workplace looks like a jail; smoking gives me the feeling that work time goes faster. Sometimes when I achieve something, I reward myself by smoking a cigarette. When patients behave well, I reward them with cigarettes. Smoking controls patients' behavior because there is nothing in this world more effective than a cigarette to control psychiatric patients who smoke. Yes, giving cigarettes to patients is used to prevent relapse and agitation (Aladdin).

At the same time, the nurses draw attention to perceived psychological and social benefits of smoking.

 The cigarette is part of my personality because I used to hold cigarettes between my fingers rather than smoke them. Now I just smoke them, which is why I smoke all the time. [A cigarette] helps my concentration during work tasks, improves my sense of freedom. I smoke to get enjoyment, to enhance my alertness, and to build a social relationship with others (...). From my perspective, smoking is necessary for psychiatric nurses because they spend the majority of their time observing patients; hence, they need to smoke to help focus their attention (Hassan).

### 3.3. Living in Ambiguity

In this second subcategory, participants reported two kinds of ambiguity that increase smoking in the workplace: the role ambiguity and the task ambiguity associated with psychiatric nursing care.

#### 3.3.1. Role Ambiguity

One of the traditional roles of JPNs is to distribute cigarettes to psychiatric patients. When the participants were asked about this role, their answers divided them into two groups. The first group of five nurses said distributing cigarettes to patients is not part of the nurse's role. Furthermore, this group of JPNs did not regard assisting patients with smoking cessation as part of their role.

 Distributing cigarettes to patients is not an official job for nurses, but we [psychiatric nurses] give patients cigarettes to stop them getting agitated. we give out cigarettes because we do not want the patients to relapse, and we want to avoid administration accusing us of not controlling patients' behaviors. As smokers ourselves, we feel empathy towards patients who smoke and we put ourselves in their shoes. cutting down on smoking is not a priority for the psychiatric patients because they are here [name of psychiatric hospital] to receive treatment for mental illness, not to quit smoking. My role as psychiatric nurse is primarily to treat psychiatric disease. I do not think that teaching patients about smoking cessation is one of my nursing roles.
 I cannot quit smoking myself, so how can I encourage others to quit? As the proverb says; a gift cannot be made of something missing. (In other words, nurses who smoke cannot be role models for changing the smoking habits of patients) (Mohammed).

The remaining three nurses thought that distributing of cigarettes to patients is a psychiatric nursing role because “I perform what administrators expect me to do (...). So, I think it is a legal role and I believe it is my job; it is a customary nursing role in the psychiatric nursing field (...). Yes, yes, it is a nursing role and part of the treatment plan the patients” (Mustafa). 

The eight JPNs were asked to describe their feelings after distributing cigarettes to patients. All eight reported ambivalent emotions in much the same way, for example:

 I feel happy because I see how patients enjoy smoking cigarettes... but at the same time, I feel guilty and disappointed because I offered them something harmful (...). Exactly what I feel, I do not know (...) I want them to smoke, but I do not like myself when give them cigarettes (...). When I distribute cigarettes I feel down because this is not my job. If there was someone else [non-nurse] to give them the cigarettes, I would be happy (Mohammed).

#### 3.3.2. Task Ambiguity/Challenges in Providing Psychiatric Nursing Care

The participants reported that the ambiguity due to the vagueness of psychiatric nursing is one of the commonest challenges they face on a daily basis. They categorized the sources of ambiguity into four levels: (1) ambiguity at the organizational and administrational level, (2) at the staff nurse level, (3) at the patient level, and (4) ambiguity of tasks at the family and societal level. These levels of ambiguity are italicized in the following participant statements.

 Psychiatric health care in Jordan is an undeveloped medical field. Much development is needed compared to other medical fields (...). Psychiatric settings in Jordan do not have a clearly organized working system (...). The big problem is that we have no multidisciplinary teams in the psychiatric field in Jordan (...). Psychiatric nurses receive low salary compared to nurses in other fields, and they have low job satisfaction (...). Indeed, in many cases, they have zero job satisfaction.... The big problem is the administrative corruption; there is cronyism and nepotism among administrators. In addition, there is an absence of trust between the administration and nurses, lack of transparency [*Ambiguity of the task at the organizational and administrational level*] (Kamal).
 Although nurses here were prepared through a Bachelor of Nursing program to provide comprehensive care, they are using the custodial model of care for psychiatric patients. They believe the patients will not respond to any treatment plan. They think that psychiatric diseases are incurable and they have accepted the role of distributing cigarettes to patients accordingly, which is completely against their health promotion role. I think all these circumstances increase my smoking rate here [name of psychiatric hospital] [*Ambiguity at the staff nurse level*] (Mohammed).
 It is horrible workload; we are two registered nurses, and three practical nurses to take care for 27 patients (...). You know, psychiatric patients often exhibit unexpected, aggressive and agitated behaviors (...). Nurses here are not sure if they can manage these behaviors (...). We are confused and upset because psychiatric patients cannot communicate effectively with us (...); they often need around-the-clock observation to prevent self-harm or harm to others (...). I am not sure if I am a nurse or a guard [*Ambiguity at the patient care level*] (Aladdin).
 Many reasons in [name of psychiatric hospital] force me to smoke more. For example, many Jordanians think that psychiatric nurses will be influenced by psychiatric patients and after a while, they [the nurses] will become crazy (...). They [Jordanians] think that mentally ill patients must always remain in hospital because mental diseases are long life and incurable. Thus, they [Jordanians] perceive psychiatric nurses as uneducated bodyguards, who protect patients from one another (...). Patients' families will do anything to keep their relatives in [name of psychiatrichospital] as long as possible (...). They [the families] do not want to take care of their relatives, but they still accuse nurses of lack of care [*Ambiguity of tasks at family and societal level*] (Hassan).

### 3.4. Experiencing Workplace Conflict

The third subcategory of *becoming a heavy smoker *is experiencing workplace conflict. This subcategory includes two conflicts reported by the participants to have increased their smoking rate: nursingrole conflict and interpersonal conflict.

#### 3.4.1. Nursing-Role Conflict

The psychiatric nurses described nursing-role conflict in the following terms.

 Nurses here [name of psychiatric hospital] are doing primarily custodial care (e.g., planning activities of daily living, administrating medications; adhering very closely to physician orders, and distributing cigarettes to patients). We do not have much authority to make decisions about treatment for the patients (...). Many of us [psychiatric nurses] reject the custodial nursing role and insist that psychiatric nursing care should be done differently based on international trends (...). I think that experiencing this conflict [in role] makes me to smoke more (Kamal).

#### 3.4.2. Interpersonal Conflict

Interpersonal conflict in the study setting arises in a variety of ways. The italicized statements below indicate a conflict between male nurses and female supervisors, among nurse coworkers, and between nurses and physicians/psychiatrists.

 I have a strongly conflicted relationship with her [a female supervisor]. The relationship can be described as fuel and fire (...) and very formal. A female leader evokes stress/creates conflicts for male followers [*Male nurses-female supervisor conflict*] (...). It is not uncommon to see some conflicts with co-workers; some of our nursing colleagues are just impossible to work with. We face difficulty and conflict when dealing with them because they are: arrogant, stubborn, sometimes abusive, slackers, spies, gossipers, and act like they are right about almost everything [*Nurse-nursing co-workers' conflict*] (Yasser).
 Physicians and nurses clash because nurses do not trust physicians and vice versa. Physicians create tensions to marginalize nurses and they ignore nursing knowledge (...). They [physicians] have misperceptions about nurses. They think nurses are not educated and they ignore nurses' requests for them [physicians] to see patients (...). They do not listen to nurses and they abuse them by shouting, by making accusations, and by blaming them [if anything goes wrong]. Nurse, use avoidance to cope with abusive physicians, thereby killing any opportunity for real communication [*Nurses-physicians conflict*] (Kamal).

### 3.5. Facing up to Workplace Stressors

This fourth subcategory of *becoming a heavy smoker *draws attention to stressors in the workplace that increase smoking among JPNs. The participants reported four sources of workplace stress: (a) the nurse does not control the steering wheel; (b) the power is within your “Wasta” (network); (c) living with negative feelings; (d) an unattractive career because due to stigmatization.

#### 3.5.1. The Nurse Does Not Control the Steering Wheel

This subcategory is characterized by limited control over decision making. One participant reported that “Jordanian psychiatric nurses cannot make any administrative decisions. The decisions they can make regarding direct patient care are very limited (...). I think that making administrative decisions is the supervisor job (...). Nurses offer opinions more than making decisions” (Kamal).

Furthermore,

 Administrators and physicians ask nurses to do only what they say and to obey their orders without question or discussion (...). They [administrators and physicians] want us to be good followers not autonomous nurses. We are here to follow orders and instructions, but we still dream to becoming decision makers in an independent profession (...). Smoking cigarettes is the most effective way to getting rid of these stressors (...) (Mohammed).

#### 3.5.2. The Power Is within Your “Wasta”/Network

*Wasta* is a popular social phenomenon in Jordan and throughout the Arab world. It can be defined simply as cronyism and corruption, but such translations do not convey either its pervasiveness or the strength of its influence.

 If anything serves as the symbol of corruption in Jordan, it is what is known as *Wasta*. Wasta literally means favouritism—the use of family, business or personal connections to advance personal interests. Although Wasta is culturally rooted, the vast majority of Jordanians believe that it is a prevalent form of corruption. At the same time, there is a public perception that citizens must have some sort of Wasta in order to run their day-to-day affairs smoothly, in a country largely ruled by bureaucracy” (Ma'ayeh, 2008) [15].

Participants reported two forms of Wasta that increases stress levels and smoking. Wasta or *cronyism at the administrational level *is manifested by “Administrators dealing with the employees on the basis of personal relationships and network ties. Feelings of injustice as a result of Wasta has increased my smoking rate (...). You know, some nurses use personal relationships to get benefits from the administrators” (Osama).

*Cronyism at the nursing supervisor level *was manifested by “Nursing supervisors deal with subordinates on the basis of personal and tribal relationships when allocating the more desired shifts [mornings] and when handling various promotions” (Aladdin).

#### 3.5.3. Intense Negative Feelings

The participants identified three causes of intense negative feelings that increase workplace stress and smoking.

*Increasing the Consumption of Cigarettes at Work. *Brought out in statements of the following kind: “We, smokers and nurses are feeling very uncomfortable as our smoking habit becomes more uncontrollable (...). We feel like we have multiple-personalities because we are nurses who smoke. We blame ourselves for our smoking. We feel guilty because we smoke” (Mohammed).

*Distributing Cigarettes to the Patients. *“I am feeling like I am cheating because I provide patients a harmful product (...). I do not feel like a nurse when I do this and this feeling is punishing me. Smoking decreases this feeling temporarily” (Aladdin).

*Lack of Control over Decision Making. *“I am feeling hopeless about never being allowed to be a decision maker (...). I am feeling as if I will always be just a follower (...). Actually, I am feeling that I have no value and I am a useless employee (...). I feel upset, burnt-out, and exhausted because I have no decision making role” (Mohammed).

#### 3.5.4. An Unattractive Career (Stigmatization)

Jordanians have misconceptions and misperceptions about psychiatric nurses because of the negative ways they [the nurses] are portrayed in the Arabic media including on TV (...). I am too worried to tell others I am a psychiatric nurse. There are very few people that I talk to about my profession (...). I do not want to talk about it [my work] because I hate feeling stigmatized because of it. Stigmatization makes us perceive nursing as a service not a profession. The nursing profession has no respect from others and has low social status in comparison to other professions (...). The stigma makes us feel pain, grief, isolation, inferiority; low self-esteem and unappreciated in our role as psychiatric nurses (...). We look at ourselves like we have limited power, knowledge, social status, and decision making (...). I smoke more and more to forget that I am working in psychiatric nursing—a low prestige profession (...). I have no job satisfaction at all and I blame myself for choosing this path (Kamal).

## 4. Discussion

Becoming a heavy smoker is the third and advanced phase in our contextualizing smoking behavior over time theory. It is a process that takes place in the work setting, a place where smoking is openly permitted and is considered to be a normal behavior. During this phase, JPNs integrate an increased rate of smoking behavior into their daily lives. This phase is similar to what DiClemente [16] called the “maintenance stage” of behavior change; it refers to when the individual becomes a fully addicted smoker, and thus smoking has become habitual and problematic. According to DiClemente [16], “the task for maintenance is to sustain and integrate the behavior change into the total life context so that it becomes normative, familiar, and integral” (page 30). A point to be emphasized regarding the difference between DiClemente's [16] “maintenance phase” and the current study is that JPNs do not “maintain” their smoking behaviors, but continue to increase their rate of smoking in the *becoming a heavy smoker *phase. Smoking behaviors are, therefore, not “maintained” but sustained at an increasingly higher rate.

### 4.1. Addiction Process

The addiction process is most commonly discussed from the perspective of two behavioral and learning theories: classical conditioning and schedules of reinforcement. From the perspective of classical conditioning, it is assumed that there is a strong relationship between smoking addictive behavior and associated stimuli [16, 17]. According to DiClemente [16], the conditioning process reaches its peak during the maintenance stage of smoking behavior. Similarly, the JPNs in out sample had many stimuli in their workplace that encouraged them to smoke with higher frequency.

As an addictive behavior, smoking is shaped by contingencies of positive and negative reinforcement [18]. JPNs who experience positive reinforcement from smoking (e.g., improved self-esteem and concentration) continue smoking to maintain positive feelings and experience other rewards as described above. However, for the nurses in out sample, the frequency of smoking is increased by negative reinforcement as well. Negative reinforcement derived from satisfying the withdrawal symptoms associated with craving more cigarettes, but it occurs also through the stress reduction that occurs as a result by smoking to escape the workplace challenges we have described. 

### 4.2. Heavy Smoking in Context

JPNs report contextual factors that influence them to become heavy smokers. The use of the constant comparison method [14] revealed that some of the findings reported here are similar to those reported in the literature, while others are unique to the current study. 

The nursing profession is distinguished from other professions by its high degree of work-related stress [19–21]. A literature review of stress among nurses showed that psychiatric nurses have a higher level of work related stresscompared with nurses in general [22]. Work-related stress occurs when the physical or psychological demands exceed the ability of employees to control their workload [23, 24]. Nursing is a stressful profession not only because it is a demanding one, but also because nurses are exposed to numerous social, physical, and environmental stressors [25]. The sources of these stressors have been identified as follows: low job control and excessive job demands [26], low control over decisions [27], and the negative leadership style of supervisors [28, 29].

Work-related stress can affect human health directly by disturbing physiological processes and indirectly through risky health behaviors such as smoking [30, 31]. Moreover, work stress affects a multitude of non-health promoting behaviors such as smoking, drinking, or weight gain more than a single unhealthy behavior [32, 33]. Previous studies indicate that many workers smoke to reduce and manage work-related stress [34–37]. For example, psychiatric nurses in the United Kingdom used smoking and alcohol to adapt to high levels of work-related stress [38].

Job satisfaction is strongly related to work-related stress. Much of the literature indicates a strong inverse relationship between job stress and job satisfaction [39–41]. For example, psychiatric nurses in the United Kingdom who had experienced a high level of stress showed low levels of job satisfaction [42]. An Australian study showed that high job satisfaction among nurses buffers and lowers work-related stress [43]. A study of the relationship between the smoking behaviors of military nurses and social support, stress, and job satisfaction found that nurses who smoked experienced a high level of work-related stress and had both low social support and low job satisfaction [44].

### 4.3. Unique Contextual Factors

In our study, JPNs reported unique contextual factors that increased their smoking rate: normalization of smoking (at nurse and organizational levels), living in ambiguity, experiencing workplace conflict, and facing up to workplace stressors, including the pernicious effect of Wasta and job stigmatization. 

## 5. Conclusion

Specific workplace contextual factors require targeted smoking reduction and smoking cessation interventions if male JPNs are to receive the help they need to reduce the health risks associated with heavy smoking for both themselves and the patients in their care.

Nurses and other decision makers can use these insights to guide culturally sensitive smoking reduction and cessation programs to benefit those male Jordanian psychiatric nurses who want to reduce their smoking or stop smoking completely.

However, smoking reduction and cessation programs are likely to be more successful in those work settings in which psychiatric nurses are encouraged to work to the full scope of their professional role.
